# Population genetic structure and natural selection of apical membrane antigen-1 in *Plasmodium vivax* Korean isolates

**DOI:** 10.1186/s12936-015-0942-6

**Published:** 2015-11-16

**Authors:** Jung-Mi Kang, Jinyoung Lee, Pyo-Yun Cho, Sung-Ung Moon, Hye-Lim Ju, Seong Kyu Ahn, Woon-Mok Sohn, Hyeong-Woo Lee, Tong-Soo Kim, Byoung-Kuk Na

**Affiliations:** Department of Parasitology and Tropical Medicine, Institute of Health Sciences, Gyeongsang National University School of Medicine, Jinju, 660-751 Republic of Korea; Department of Tropical Medicine, Inha Research Institute for Medical Sciences, Inha University School of Medicine, Incheon, 400-712 Republic of Korea; Department of Internal Medicine, Seoul National University Bundang Hospital, Seongnam, 463-707 Republic of Korea; Department of Pathology, Immunology, and Laboratory Medicine, College of Medicine, University of Florida, J-566, 1275 Center Drive, Gainesville, FL 32610 USA

**Keywords:** *Plasmodium vivax*, Apical membrane antigen-1, Vaccine, Genetic polymorphism, Natural selection, Korea

## Abstract

**Background:**

*Plasmodium vivax* apical membrane antigen-1 (PvAMA-1) is a leading candidate antigen for blood stage malaria vaccine. However, antigenic variation is a major obstacle in the development of an effective vaccine based on this antigen. In this study, the genetic structure and the effect of natural selection of PvAMA-1 among Korean *P. vivax* isolates were analysed.

**Methods:**

Blood samples were collected from 66 Korean patients with vivax malaria. The entire PvAMA-1 gene was amplified by polymerase chain reaction and cloned into a TA cloning vector. The PvAMA-1 sequence of each isolate was sequenced and the polymorphic characteristics and effect of natural selection were analysed using the DNASTAR, MEGA4, and DnaSP programs.

**Results:**

Thirty haplotypes of PvAMA-1, which were further classified into seven different clusters, were identified in the 66 Korean *P. vivax* isolates. Domain II was highly conserved among the sequences, but substantial nucleotide diversity was observed in domains I and III. The difference between the rates of non-synonymous and synonymous mutations suggested that the gene has evolved under natural selection. No strong evidence indicating balancing or positive selection on PvAMA-1 was identified. Recombination may also play a role in the resulting genetic diversity of PvAMA-1.

**Conclusions:**

This study is the first comprehensive analysis of nucleotide diversity across the entire PvAMA-1 gene using a single population sample from Korea. Korean PvAMA-1 had limited genetic diversity compared to PvAMA-1 in global isolates. The overall pattern of genetic polymorphism of Korean PvAMA-1 differed from other global isolates and novel amino acid changes were also identified in Korean PvAMA-1. Evidences for natural selection and recombination event were observed, which is likely to play an important role in generating genetic diversity across the PvAMA-1. These results provide useful information for the understanding the population structure of *P. vivax* circulating in Korea and have important implications for the design of a vaccine incorporating PvAMA-1.

## Background

*Plasmodium vivax* is the most prevalent human malaria parasite globally and is responsible for a large proportion of the global malaria burden, especially in regions outside of Africa [1]. Although it has been neglected as a benign infection, *P. vivax* causes serious clinical illnesses including respiratory distress, severe anemia, coma and even death [2, 3]. Moreover, it has recently re-emerged in many temperate regions from where it had been largely eradicated during global malaria control campaigns. The emergence of drug resistance strains also complicates the burden of the parasite [4, 5]. Considering the enormous socio-economic impact of *P. vivax* on humans, development of an effective vaccine is an important concern in control and elimination strategies. However, no effective vaccine is yet available and the antigenic diversity present in wild-type isolates, which has led to the failure of several licensed and tested malaria vaccines, has been recognized as a major concern in developing a successful vaccine.

Apical membrane antigen-1 (AMA-1) is a type 1 integral membrane protein that is expressed in the late schizont stage of malaria parasites [6]. It is initially synthesized in the micronemes of the apical complex of merozoites and is transported to the surface of parasite just prior to erythrocyte invasion, where it undergoes proteolytic cleavage [7, 8]. Although the precise role of AMA-1 is not fully understood, it is believed to be essential in erythrocyte invasion [9]. AMA-1 consists of a signal sequence, a cysteine-rich ectodomain, a conserved cytoplasmic region and a transmembrane region. The ectodomain is further divided into three distinct domains (domains I, II and III) by disulfide bridges [10]. The ectodomain of AMA-1 is very immunogenic and a high antibody titer against the domain is produced in humans who are naturally infected with malaria parasites [11–14]. Antibodies against AMA-1 effectively inhibit erythrocyte invasion [15–17]. Therefore, AMA-1 has been considered as a promising candidate antigen for blood stage malaria vaccine [18, 19].

Although it is recognized that AMA-1 is less variable than the other blood stage malaria vaccine candidate antigens such as merozoite surface protein antigens (MSPs) and circumsporozoite protein (CSP), it also shows sequence variations among global malaria parasites [20–23]. In *P. falciparum*, most of the genetic polymorphisms are concentrated in domain I [20, 21, 24], while the majority of the polymorphic patterns of AMA-1 occur in domains I and II in *P. vivax* [22–27]. These polymorphisms result in amino acid changes in the natural population, indicating that PvAMA-1 is under natural selection, may be a result of host immune pressure [23, 26].

In this study, the population genetic structure and natural selection of PvAMA-1 among Korean *P. vivax* isolates was analysed. A higher rate of polymorphic patterns and evidence of natural selection were identified in domains I and III. The Korean PvAMA-1 showed different polymorphic patterns compared to other global isolates. Recombination also likely has been important in generating genetic diversity across the PvAMA-1 sequences. These results provide useful information for the understanding of the population structure of *P. vivax* circulating in Korea and have important implications for the design of a vaccine incorporating PvAMA-1.

## Methods

### Blood samples and study areas

A total of 66 blood samples from Korean patients with uncomplicated *P. vivax* were collected at endemic areas between 2009 and 2011. The *P. vivax* infection was confirmed by microscopic examination of thin and thick blood smears and species-specific polymerase chain reaction (PCR) [28]. All the patients resided in malaria endemic regions, northwestern part of Gyeonggi province near DMZ in Korea, and have not been abroad at least in past 2 years when their blood samples were collected. The collected blood was separated into packed cells and plasma and then stored at −80 °C until use. Informed consent was obtained from all of the patients before blood collection. The study protocol was approved by the Ethics Committee of the Inha University School of Medicine.

### Amplification and sequencing analysis of the PvAMA-1

Genomic DNA of parasite was extracted from 200 µl of whole blood sample using the QIAamp DNA Blood Kit (Qiagen, Hilden, Germany). The entire PvAMA-1 gene was amplified using two rounds of PCR. The primers were designed based on conserved regions of PvAMA-1 sequences of Sal I (AF063138) and Belem (EU395595). The forward and reverse primers used for the first round of PCR were 5′-GCAGAGAGAGCAAACCAAATCG-3′ and 5′-GCAAGCGAGTTGGCCAAGCAAA-3′. The primers used for the nested PCR were 5′-ATGAATAAAATATACTACATAATCTTTTTA-3′ and 5′-TTAGTAGTATGGCTTCTCCATCAG-3′. The following thermal cycling conditions were used for both amplifications: 94 °C for 5 min; 30 cycles of 94 °C for 1 min, 52 °C for 1 min and 72 °C for 2 min, and a final extension at 72 °C for 10 min. To minimize the nucleotide mis-incorporation in the sequences during PCR amplification, Ex Taq DNA polymerase (Takara, Otsu, Japan), which has a proofreading activity, was used in all PCR process. Each resulting PCR product was resolved on a 1.2 % agarose gel, purified from the gel, and ligated into the T&A cloning vector (Real Biotech Corporation, Banqiao City, Taiwan). Each ligation mixture was transformed into *Escherichia coli* DH5α competent cells and positive clones with the appropriate insert were selected by colony PCR. The nucleotide sequences of the cloned insert were analysed by automatic DNA sequencing with M13 forward and M13 reverse primers. Sequencing analyses with two additional specific internal primers (5′-GAAGTTAAACGATATAGCTTTGTGCAG-3′ and 5′-TCAACACTGTACAGATTCATGTTCCTC-3′) were also performed to obtain the sequences of central region of PvAMA-1. At least two clones from each isolate were sequenced to ensure sequencing accuracy, and some isolates underwent three- or four-fold sequence coverage to confirm the existence of rare polymorphisms. The nucleotide sequences reported in this study have been deposited in the GenBank database under the accession numbers (KM230319–KM230384).

### Sequence and phylogenetic analysis of PvAMA-1

The nucleotide and deduced amino acid sequences of PvAMA-1 were analysed using EditSeq and SeqMan in the DNASTAR package (DNASTAR, Madison, WI, USA). The phylogenetic tree was constructed using the neighbour-joining method in MEGA4 computational program [29]. Bootstrap proportions were used to assess the robustness of the tree with 1000 bootstrap replicates.

### Sequence analysis

DNA sequence polymorphism analysis was performed on the 66 PvAMA-1 sequences. The number of segregating sites (S), haplotypes (H), haplotype diversity (Hd), nucleotide diversity (π), and the average number of pair-wise nucleotide differences within the population (*K*) were estimated using the DnaSP ver. 5.10.00 [30]. The π was calculated to estimate the step-wise diversity throughout the entire PvAMA-1 based on a sliding window of 100 bases with a step size of 25 bp. The rates of synonymous (dS) and non-synonymous (dN) substitutions were estimated and were compared using the Z test (P < 0.05) in MEGA4 program [29] using Nei and Gojobori’s method [31] with the Jukes and Cantor correction. To evaluate the neutral theory of evolution, the Tajima’s D value [32] and Fu and Li’s D and F statistics [33] were analysed using the DnaSP ver. 5.10.00 [30].

### Recombination parameters and linkage disequilibrium

The recombination parameter (R), which included the effective population size and probability of recombination between adjacent nucleotides per generation, and the minimum number of recombination events (Rm) were determined using the DnaSP ver. 5.10.00 [30]. The linkage disequilibrium (LD) between the different polymorphic sites was computed based on the R^2^ index.

## Results

### Sequence polymorphism in Korean PvAMA-1

**Fig. 1:**
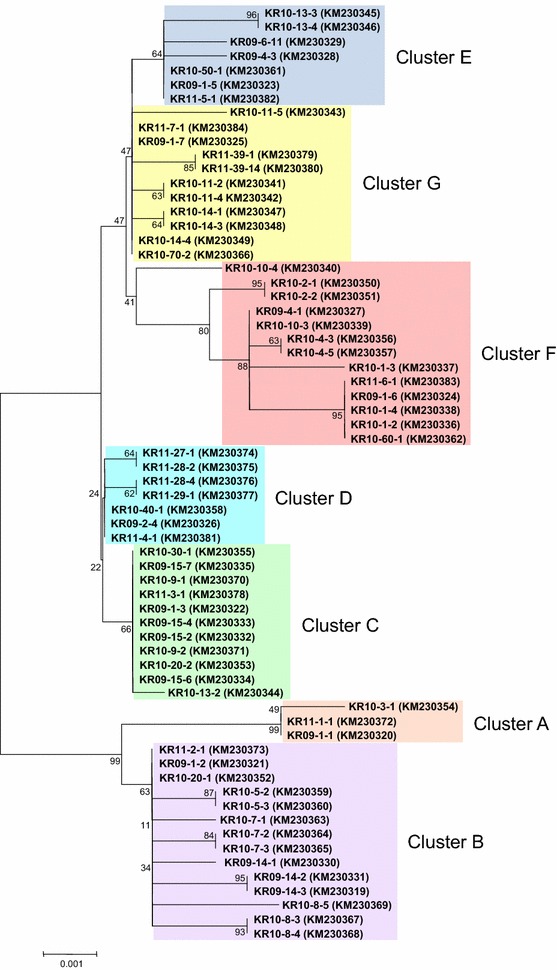
Phylogenetic analysis of the PvAMA-1 sequences. The tree was constructed with 66 PvAMA-1 sequences obtained from Korean *P. vivax* isolates using a neighbour-joining method. The 66 sequences were divided into seven distinct clusters (*cluster A*–*G*). The tree was constructed using 1000 bootstrap replicates

**Fig. 2:**
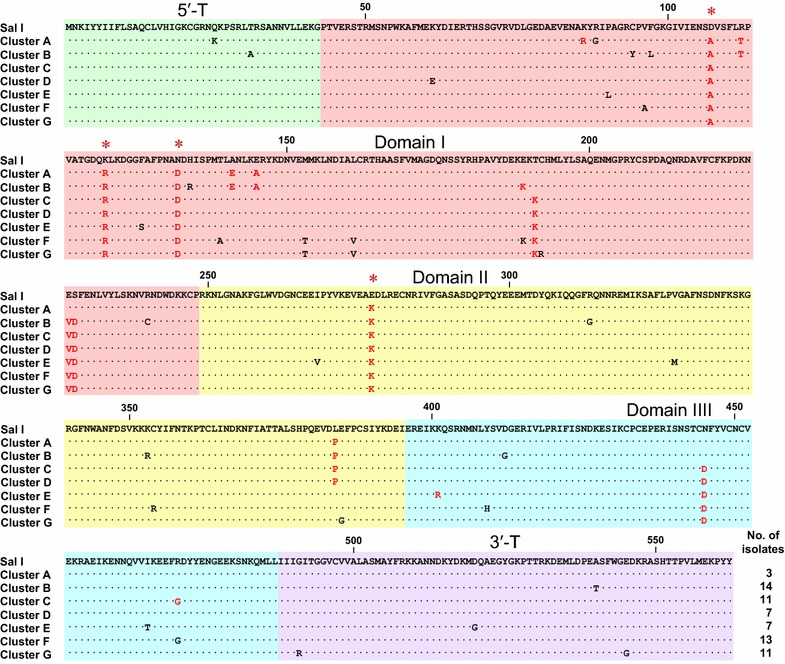
Sequence polymorphism of the PvAMA-1 in Korean *Plasmodium vivax* isolates. The amino acid sequences of Korean PvAMA-1 were compared to the Sal I sequence (AF063138). Identical amino acid residues are indicated by *dots*. The 44 non-synonymous SNPs identified in 66 Korean PvAMA-1 resulted in 44 amino acid changes, all of which were dimorphic. The amino acid changes were not evenly distributed in each PvAMA-1 haplotype, but the sequences clustered into an individual cluster share common amino acid changes. The conserved amino acid changes identified in all sequences in the corresponding cluster were represented as *red* and amino acid changes identified in at least one sequence in the corresponding cluster were marked as *black*. *Red asterisks* indicate the commonly identified amino acid changes in all Korean PvAMA-1. The total number of isolates for each cluster is listed in the *right panel*

Sixty-six PvAMA-1 sequences with 30 different haplotypes were obtained from Korean *P. vivax* isolates. Phylogenetic analysis of the sequences classified the 66 Korean PvAMA-1 sequences into seven distinct clusters (clusters A–G; Fig. 1). Nucleotide sequence analysis of the 66 sequences compared to *Sal*I (AF063138) revealed 66 single nucleotide polymorphisms (SNPs) in the Korean PvAMA-1 sequences. The 22 SNPs were synonymous and the others were non-synonymous. The non-synonymous SNPs resulted in 44 dimorphic amino acid changes in 66 PvAMA-1 sequences (Fig. 2). Although these amino acid changes were not evenly distributed in each PvAMA-1 haplotype, four amino acid changes (D107A, K120R, N132D and E277K) were commonly found in all PvAMA-1 sequences (Fig. 2). L384P was commonly identified in the sequences in clusters A, B, C and D. E227V and S228D were highly conserved in sequences belonging to clusters B, C, D, E, F and G. T191K and N445D were also highly conserved in sequences in clusters C, D, E, F and G. The other amino acid changes were randomly distributed in each haplotype or were conserved among the sequences that were classified into the same cluster. For example, R112T was identified only in the sequences clustered into clusters A and B. Meanwhile, K86R, E189K, R471G and K401R were conserved in cluster A, B, C and E, respectively.

These amino acid changes were mainly identified in domain I, but with significant rates in domains II and III. Six amino acid changes (Q25K, T31A, G491R, D520G, A540T and E545G) were found in the 5ʹ-terminal (5ʹ-T) or 3ʹ-terminal (3ʹ-T) region. Among the 44 dimorphic amino acid changes, 15 have been previously reported. The remaining 29 changes (Q25K, T31A, K61E, K86R, R88G, P90L, C94Y, V96A, F97L, H134R, T139A, M153T, L161V, T191K, C192R, R240C, I268V, R313G, V327M, K353R, C354R, K401R, D412G, I466T, R471G, G491R, D520G, A540T and E545G) are novel ones that have not been reported previously.

### Comparison of amino acid polymorphisms of Korean PvAMA-1 with global *P. vivax* isolates

**Fig. 3:**
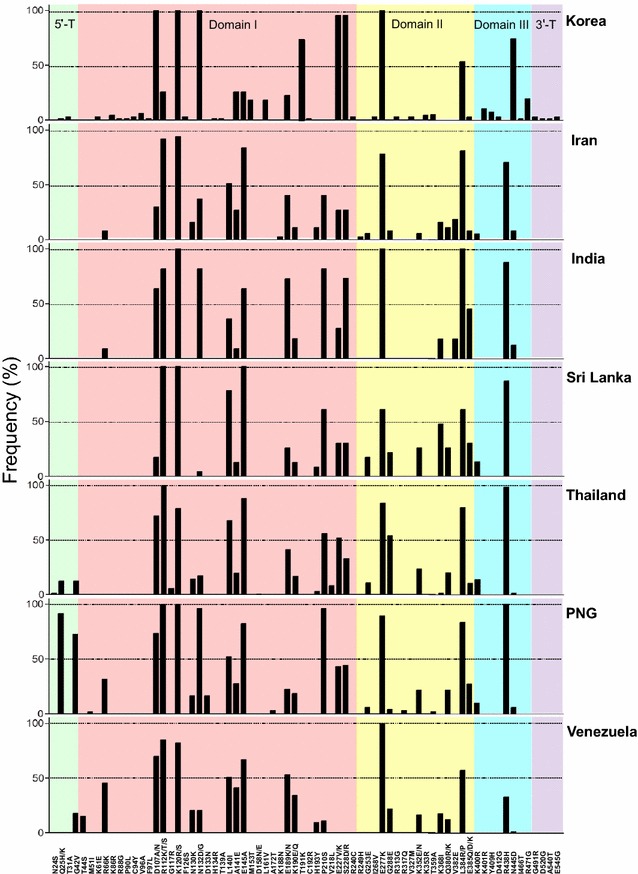
Comparison of amino acid polymorphisms of PvAMA-1 among global *Plasmodium vivax* isolates. The frequencies of amino acid changes found in PvAMA-1 in *P. vivax* isolated in different geographical regions

Korean PvAMA-1 showed different patterns of amino acid polymorphisms compared to those from other geographical regions (Fig. 3). The most significant characteristic in Korean PvAMA-1 was that four amino acid changes (D107A, K120R, N132D and E277K) were tightly conserved in all 66 PvAMA-1 sequences. E227V and S228D (63/66; 95.5 %, respectively), T191K and N445D (49/66; 74.2 %, respectively) and L384P (35/66; 53.03 %) also showed high level of polymorphic patterns. High frequency of D107A was also identified in PvAMA-1 sequences from India (63.6 %), Thailand (72.2 %), Papua New Guinea (PNG, 73.5 %) and Venezuela (69.9 %), but its frequency was low in the sequences from Iran (29.7 %) and Sri Lanka (17.4 %). K120R and E277K showed high level changes in the global PvAMA-1 sequences currently analysed. N132D was very frequent in India (81.8 %) and PNG (96.1 %), but was less than 50 % in Iran, Sri Lanka, Thailand and Venezuela. E227V and S228D changes were tightly linked in Korean PvAMA-1 sequences. Interestingly, T191K and N445D, which were identified in high levels of frequency in Korean PvAMA-1, were not or rarely identified in PvAMA-1 sequences from other geographical areas. Meanwhile, R112K/T/S, L140I, E145A, P210S and R438H, which were highly polymorphic in PvAMA-1 sequences from other geographical areas, were not identified or detected at low levels in Korean PvAMA-1.

### Nucleotide diversity and natural selection

**Fig. 4:**
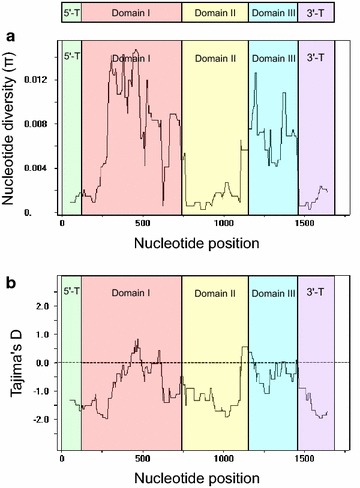
Nucleotide diversity and natural selection of PvAMA-1 in Korean isolates. **a** Nucleotide diversity. Sliding window analysis showed nucleotide diversity (π) values in 66 PvAMA-1 sequences analysed. A window size of 100 bp and a step size of 25 bp were used. **b** Natural selection. Sliding window calculation of Tajima’s D statistic was performed for the 66 PvAMA-1 sequences. A window size of 100 and a step size of 25 were used

DNA sequence analyses were conducted to determine nucleotide diversity and genetic differentiation of PvAMA-1 in the Korean *P. vivax* isolates. The *K* value for the 1687 bp of full length PvAMA-1 was 8.07 (Table 1). The highest nucleotide differences was identified at domain I (*K* = 4.856) and was the lowest at 5ʹ-T. The Hd for the entire PvAMA-1 sequences of 66 isolates was 0.961 ± 0.012. This value was higher in domain III than in domains I and II. The π of the entire PvAMA-1 for the 66 Korean isolates was 0.00478 ± 0.00038. Analysis of the π for the 5ʹ-T, domain I, domain II, domain III and 3ʹ-T indicated that most of the nucleotide diversity was concentrated in domains I and III. The π value for each fragment was 0.00071 ± 0.00039 (5ʹ-T), 0.00786 ± 0.00087 (domain I), 0.00240 ± 0.00030 (domain II), 0.00601 ± 0.00054 (domain III) and 0.00132 ± 0.00048 (3ʹ-T) (Table 1). Overall nucleotide diversity observed across the entire PvAMA-1 indicated peak nucleotide diversity in domains I and III (Fig. 4a). To examine whether natural selection contributed to generation of the diversity in PvAMA-1 of the Korean *P. vivax* population, the rate of dN to dS was estimated using the Nei and Gojobori’s method [30]. The dN (0.00379) exceeded dS (0.00541), and the dN/dS ratio of 0.703 suggested that negative natural selection may be occurring in the PvAMA-1 of Korean *P. vivax* isolates (Table 1). To further analyse the natural selection occurs in PvAMA-1 of Korean *P. vivax* population, Tajima’s D test was performed. The estimated value was −1.4140 (P > 0.10), indicating that PvAMA-1 is under population size expansion and/or purifying selection pressure (Table 1). The Fu and Li’s D and F values for the entire PvAMA-1 were also negative: −1.5383 (P > 0.10) and −1.2030 (P > 0.10), respectively (Table 1). Tajima’s D values for each fragment of PvAMA-1 also showed negative values: 5ʹ-T (−1.3151, P > 0.10), domain I (−1.1028, P > 0.10), domain II (−1.5941, 0.05 < P < 0.10), domain III (−0.5681, P > 0.10) and 3ʹ-T (−1.9993, P < 0.05). However, the middle region of domain I and the junction between domains II and III showed positive Tajima’s D values (Fig. 4b), indicating that the regions could be a dominant target of host immune response.

**Table 1:** Estimates of DNA sequence polymorphism and tests of neutrality at PvAMA-1 among *Plasmodium vivax* Korean isolates

Fragment	Segregating sites (S)	Singleton variable sites	Parsimony informative sites	Total no. of mutations	*K*	H	Hd ± SD	π ± SD	dN/dS	Tajima’s D	Fu and Li’s D	Fu and Li’s F
5ʹ-Terminal	2	1	1	2	0.090	3	0.089 ± 0.048	0.00071 ± 0.00039	0	−1.3151 (P > 0.10)	−1.2487 (P > 0.10)	−0.9681 (P > 0.10)
Domain I	35	10	25	35	4.856	20	0.830 ± 0.043	0.00786 ± 0.00087	0.765	−1.1028 (P > 0.10)	−0.9845 (P > 0.10)	−0.6579 (P > 0.10)
Domain II	11	4	7	11	0.985	10	0.682 ± 0.038	0.00240 ± 0.00030	1.946	−1.5941 (0.05 < P <0.1)	−1.4316 (P > 0.10)	−1.0111 (P > 0.10)
Domain III	11	3	8	11	1.839	12	0.848 ± 0.023	0.00601 ± 0.00054	0.433	−0.5681 (P > 0.10)	−0.5407 (P > 0.10)	−0.3995 (P > 0.10)
3ʹ-Terminal	7	4	3	7	0.300	6	0.201 ± 0.066	0.00132 ± 0.00048	0.358	−1.9993 (P < 0.05)	−2.3973 (P < 0.05)	−2.0530 (P < 0.05)
Full	66	22	44	66	8.070	30	0.961 ± 0.012	0.00478 ± 0.00038	0.703	−1.4140 (P > 0.10)	−1.5383 (P > 0.10)	−1.2030 (P > 0.10)

*K* average number of pairwise nucleotide differences, *H* number of haplotypes, *Hd* haplotype diversity, *π* observed average pairwise nucleotide diversity, *dN* rate of non-synonymous mutations, *dS* rate of synonymous mutations

### Recombination and linkage disequilibrium

**Fig. 5:**
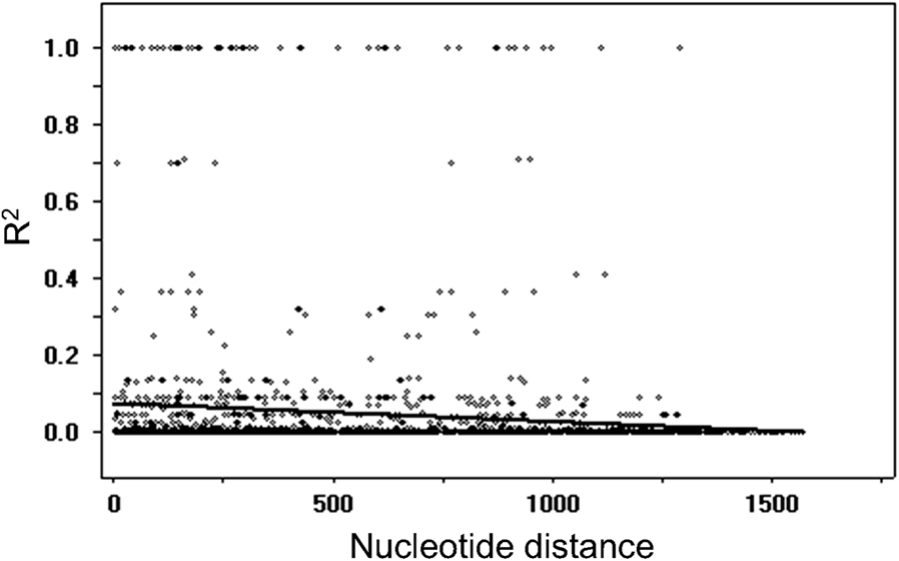
Recombination event in PvAMA-1. The linkage disequilibrium (LD) plot showed non-random associations between the nucleotide variants in 66 Korean PvAMA-1 sequences at different polymorphic sites. The R^2^ values were plotted against nucleotide distance using a two-tailed Fisher’s exact test for statistical significance

For Korean PvAMA-1, the minimum number of recombination events between adjacent polymorphic sites (Rm) was estimated as 3, whereas the R between adjacent site (Ra) and per gene (Rb) was 0.0056 and 9.5, respectively. The plausible recombination sites were predicted to be localized in domain I and domain III. These high values of the recombination parameters suggested that meiotic recombination may occur between sites, resulting in genetic diversity of PvAMA-1. The LD index (R^2^) also declined across PvAMA-1, suggesting that intragenic recombination may also be contributing the PvAMA-1 diversity (Fig. 5).

## Discussion

Vivax malaria re-emerged in Korea in 1993 near the demilitarized zone (DMZ) border with North Korea [34]. Since its re-emergence, the outbreak has spread into cities and counties adjacent to the DMZ and has persisted until now with fluctuating numbers of annual indigenous cases, with 32,300 official cases [35]. Understanding the nature of the genetic population of *P. vivax* circulating in Korea is beneficial to knowledge of the nationwide parasite heterogeneity and in the implementation malaria control programs in the country. Moreover, this information is helpful for the development of an effective malaria vaccine. Several studies on genetic diversity of polymorphic marker proteins including MSP-1, MSP-3α, MSP-3β and CSP in Korean *P. vivax* suggested that rapid dissemination of genetic structure of *P. vivax* is processed in recent years [36–42]. Characterization of genetic property of PvAMA-1 in Korean *P. vivax* isolates has also been analysed [43, 44], but these studies analysed sequence polymorphism restricted to partial region corresponding domain I and not the entire PvAMA-1. In the present study, the genetic polymorphisms and natural selection in the entire PvAMA-1 among *P. vivax* Korean isolates were analysed to gain an in-depth understanding of the nature of PvAMA-1 in Korean *P. vivax* population.

The 66 PvAMA-1 sequences obtained from Korean *P. vivax* isolates were classified into 30 different haplotypes, which were further clustered into seven distinct clusters. Compared to Sal I sequence, 66 SNPs, which resulted in 44 dimorphic amino acid substitutions, were identified in Korean PvAMA-1. The majority of the amino acid changes clustered within PvAMA-1 domain I, as has been reported previously for *P. vivax* and *P. falciparum* AMA-1 [20–23, 26, 27, 45]. The 15 amino acid changes identified in Korean PvAMA-1 are comparable to those previously identified [22, 23, 26, 27], which suggests that the polymorphic sites are under high natural selection. The 29 amino acid changes were novel and have not been reported, even though they were detected with low frequencies in Korean PvAMA-1. Compared to previously reported North Korea (EU395599) and South Korea (SK0814; GU476844) sequences, which are only two entire PvAMA-1 sequences currently available in Korean Peninsula-origin *P. vivax*, Korean PvAMA-1 sequences analysed in this study shared similar patterns of amino acid changes. All amino acid changes reported in the North Korea and South Korea sequences were commonly identified in all Korean PvAMA-1 sequences analysed currently. In particular, the four tightly conserved amino acid changes (D107A, K120R, N132D and E277K), which are the most outstanding characteristic found in Korean PvAMA-1 analysed in this study, were well-conserved in the all the sequences. Based on the sequence polymorphism analyses of the current study, the North Korea sequence was most similar to the sequences belongs to cluster A, while the South Korea sequence was more closely related with the sequences of cluster D or G. Interestingly, Korean PvAMA-1 showed different patterns of polymorphism compared to previously reported sequences from other geographical regions [22–27]. Although K120R/S and E277K were also identified at high frequencies (up to 50 %) in PvAMA-1 from other different geographical areas, D107A/N frequency was low in Iran (29.7 %) and Sri Lanka (17.4 %) and the N132D/G frequency was low in Iran (37.0 %), Sri Lanka (4.3 %), Thailand (17.1 %) and Venezuela (20.6 %) [22–27]. S228D, which showed a high frequency (95.5 %) in Korean PvAMA-1, was identified with a high frequency only in Indian isolates (72.8 %) [25]. Meanwhile, the amino acid changes (R112K/T/S, L140I, E145A, P201S and R438H), which showed high frequencies in other geographical regions, were not identified or detected only with low levels of frequency in Korean PvAMA-1. Unlike the relatively even global distribution of PfAMA-1 diversity [46, 47], substantial geographical differentiation between populations was observed for PvAMA-1 [23]. The results of this study also suggest that Korean PvAMA-1 showed different patterns of polymorphic nature compared to those of other geographical areas and substantial geographic differentiation was observed among global isolates.

Several studies have suggested that the most common polymorphic amino acid residues identified in PvAMA-1 are located on one face of the protein, which suggests that this face is more exposed to the exterior environment and is accessible to host immune responses [21, 23, 27, 48]. Consistent with the previous studies, the majority of SNPs detected in Korean PvAMA-1 are exposed on one face of the protein. A recent study on the epitope mapping prediction of PvAMA-1 suggested that the potential B cell epitopes across the ectodomain [27]. Some of the SNPs identified in global *P. vivax* isolates, including E145K, P210S, R249H, G253E, K352E, R438H and N445D, overlap with the B-cell epitope regions. These amino acid changes may affect the protein structure by causing changes in charge and polarity of the protein and might help parasites to escape from host protective immunity [27]. No or few changes were observed in the corresponding amino acids in Korean PvAMA-1, but N445D was identified with a high frequency (74.2 %), which differs significantly from isolates from other different geographical areas. Three additional amino acid substitutions (Q380K, V382E and L384P/R) were also may alter protein structure by causing changes in protein polarity and hydrophilicity [27], which might decrease epitope binding scores or loss of the predicted linear B cell epitope either together or in combination with proximal polymorphisms [49]. Q380K and V382E were not identified in Korean PvAMA-1, but L384P was observed at a high frequency (53.3 %). Intrinsically unstructured/disordered regions (IURs), which are widely identified in eukaryotic proteins, play important roles in many fundamental cell functions such as molecular recognition, molecular assembly and protein modification [50, 51]. *Plasmodium* spp. also have many proteins containing IURs and these proteins have been considered important in attachment and invasion of the parasite into red blood cells, although their specific functions remain to be defined [52]. Thereby overlap region of B-cell epitopes and IURs has been postulated more likely to represent a real antigenic/immunogenic region within a protein and the co-occurrence of both regions increases the chance of presenting this region with no secondary structure [52, 53]. The predicted overlap IUR and B-cell epitope, which comprises 18 amino acid residues (SASDQPTQYEEEMTDYQK), is highly antigenic during natural human infections and is an important antigenic region of the domain II of PvAMA-1 [27, 53]. This linear epitope was highly conserved in all Korean PvAMA-1 sequences, as it was strictly conserved in currently analysed global isolates [27]. This suggests a high degree of amino acid sequence conservation of the region among global *P. vivax* isolates and further supports the hypothesis that these amino acid sequences in domain II are subjected to strong purifying selection that might be used as a component of a PvAMA-1-based vaccine.

The level of nucleotide diversity at entire PvAMA-1 in Korean *P. vivax* isolates (π = 0.00478) was relatively lower than those for isolates from different geographical areas, including Iran (π = 0.00826), Sri Lanka (π = 0.00675), PNG (π = 0.0079), Venezuela (π = 0.0065) and Thailand (π = 0.0089) [22, 23, 27]. Lower malaria transmission in Korea compared to other endemic countries may contribute to the lower genetic diversity observed in Korean PvAMA-1. These collective results are suggestive of a low level of genetic polymorphism occurring at Korean PvAMA-1. The nucleotide diversity was not evenly distributed across entire PvAMA-1. The 5ʹ-T and 3ʹ-T showed low levels of nucleotide diversity, which suggests that the two regions are relatively well conserved in Korean PvAMA-1. A high level of nucleotide diversity was observed in domain I and domain III in PvAMA-1 of Korean *P. vivax* isolates, different from previous reports that nucleotide diversity is highest in domains I and II of PvAMA-1 in Indian, Sri Lankan and Thailand *P. vivax* isolates [22, 26, 27]. This indicates that domain II is more conservative with lower frequency polymorphisms than domains I and III in Korean PvAMA-1, even though a cluster of SNPs was observed in domain II. Natural selection affected by host immune responses and recombination between genetically distinct malaria parasites during meiotic replication in the mosquito midgut have been recognized as the two main mechanisms by which PvAMA-1 genetic diversity is generated and maintained [49]. The dN/dS for the entire length Korean PvAMA-1 was estimated to 0.703, suggesting that purifying selection pressure may act on the protein [54]. However, the dN/dS value of domain II was higher than 1 (1.946), which implies that positive natural selection may be occurring in the domain. Negative values of Tajima’s D (−1.4140), Fu and Li’s D (−1.5383) and Fu and Li’s F (−1.2030) for entire Korean PvAMA-1 presently analysed imply an excess of low frequency polymorphisms indicating population size expansion and/or purifying selection [31]. Each domain of Korean PvAMA-1 also showed negative values of Tajima’s D, Fu and Li’s D and Fu and Li’s F. These results clearly point to stronger diversifying selection, probably by host immune selection pressure, is working at Korean PvAMA-1. Meiotic recombination that occurs between the adjacent polymorphic sites drives allelic diversity of PvAMA-1 [22, 26]. Evidence of recombination event within the Korean PvAMA-1 was also observed, which supported by the decline of LD index R^2^ with increasing nucleotide distance, as consistent with the previous studies.

## Conclusions

A major concern in the development of effective malaria vaccine is genetic polymorphisms observed among global isolates. PvAMA-1 is one of the most promising candidates for malaria vaccine targeting the blood stages of *P. vivax*. Genetic investigation of Korean PvAMA-1 suggests that this antigen shows a limited antigenic diversity, but most of the major amino acid polymorphisms located on one face of the protein were commonly identified among worldwide *P. vivax* isolates. However, the overall pattern of genetic polymorphism of Korean PvAMA-1 slightly differed from those from other geographical isolates. Moreover, 29 novel polymorphic changes, most of which are predicted to be located outside B-cell epitopes, were also observed in Korean PvAMA-1. Genetic diversity of Korean PvAMA-1 was relatively lower than currently analyzed PvAMA-1 from other geographical areas, but evidences for natural selection and recombination were found. These results have significant implications in understanding the nature of Korean *P. vivax* population and in providing useful information for malaria vaccine development based on this antigen.
